# Assessing the Prognostic Capability of Immune-Related Gene Scoring Systems in Lung Adenocarcinoma

**DOI:** 10.1155/2022/2151396

**Published:** 2022-07-31

**Authors:** Wenhao Liu, Ruihong Dong, Shuai Gao, Xiaodi Shan, Mian Li, Zhaoyan Yu, Liang Sun

**Affiliations:** ^1^College of Artificial Intelligence and Big Data For Medical Sciences, Shandong First Medical University & Shandong Academy of Medical Sciences, Jinan, Shandong, China; ^2^Beijing Mentougou Hospital of Traditional Chinese Medicine, Beijing, China; ^3^Department of Rehabilitation, Beijing Rehabilitation Hospital of Capital Medical University, Beijing, China; ^4^First Affiliated Hospital of Shandong First Medical University, Biomedical Sciences College & Shandong Medicinal Biotechnology Centre, Shandong First Medical University & Shandong Academy of Medical Sciences, Jinan, Shandong 250000, China; ^5^Department of Otorhinolaryngology, Shandong Public Health Clinical Center, Jinan, Shandong, China

## Abstract

**Background:**

Lung adenocarcinoma (LUAD) is the commonest of the subtypes of lung cancer histologically. For this study, we intended to analyze the expression profiling of the immune-related genes (IRGs) from an independently available public database and developed a potent signature predictive of patients' prognosis.

**Methods:**

Gene expression profiles and the clinical data of lung adenocarcinoma were gathered from the Gene Expression Omnibus database (GEO) and The Cancer Genome Atlas (TCGA), and the obtained data were split into a training set (*n* = 226), test set (*n* = 83), and validation set (*n* = 400). IRGs were then gathered from the ImmPort database. A prognostic model was constructed by analyzing the training set. Then the GO and KEGG analysis was performed, and a gene correlation prognostic nomogram was constructed. Finally, external validation, such as immune infiltration and immunohistochemistry, was performed.

**Results:**

The 110 genes were significant by univariate Cox regression analysis and randomized survival forest algorithm for the training set and showed a good distinction between the low-risk-score and high-risk-score groups in the training set (*P* < 0.0001) by screening for four prognosis-related genes (HMOX1, ARRB1, ADM, PDIA3) and validated by the test set GSE30219 (*P*=0.0025) and TCGA dataset (*P*=0.00059). Multivariate Cox showed that the four gene signatures were an individual risk factor for LUAD. In addition, the genes in the signatures were externally verified using an online database. In particular, PDIA3 and HMOX1 are essential genes in the prognostic nomogram and play an important role in the model of immune-related genes.

**Conclusion:**

Four immune-related genetic signatures are reliable prognostic indicators for patients with LUAD, providing a relevant theoretical basis and therapeutic rationale for immunotherapy.

## 1. Introduction

Lung cancer is major cancer in the world and adenocarcinoma patients lack tumor-specific clinical symptoms in the early stage, and local infiltration and even distant metastasis occur in the middle and late stages of lung cancer, with poorer efficacy and overall survival rate. Currently, 60% of NSCLC is LUAD, of which NSCLS is the major component of lung cancer [1, 2]. In the United States, in 2021, the number of new incidences in a year was 235,760, and the number of deaths reached 131,880 [3]. For treating patients with early LUAD, surgical lobectomy is the most frequently used method. However, 10–44% of them have a less favorable prognosis five years after surgery [4]. Therefore, it is necessary to develop more effective biomarkers to obtain more effective prognostic models of LUAD patients to help early diagnosis of LUAD and treat different patients with reasonable treatment plans so that patients can receive more appropriate treatment for their own conditions and get the best treatment results.

It is generally accepted that cancer is an incredibly complex disease that involves the interplay between the tumor and the immune system [5]. The human immune-related systems have been confirmed to play a critical part in the development and progression of aggressive cancer [6, 7]. More recently, immune checkpoint blockade therapy has shown remarkable efficacy in treating solid tumors (melanoma, lung cancer, and so on). This treatment is based on a dynamic process between tumor and immunity, and most current studies predict efficacy based on marker levels before or at a point in time during treatment. Programmed death-ligand (PD-1) and tumor mutation burden (TMB) are widely used as prognostic biomarkers for immunotherapy [8]. However, immune-related therapies are only available for some patients. There are remarkable personal differences in therapeutic effectiveness, illustrating the complexity of cancer-causing mechanisms and the existence of tumor heterogeneity [9, 10]. A few studies have reported that immune-related genes can predict prognosis in tumor survivors and provide potential targets for immunotherapeutic treatment [11–13]. However, the prognostic model of immune-related genes in early-stage LUAD patients is relatively rare.

This study explores early LUAD-related prognostic models based on immune-related genes based on gene expression datasets from GEO and TCGA. After we matched the immune-related genes list to the three cohorts, the resulting genes were screened by a series of methods. An immune signature based on four genes for prognosis with outstanding predictive capability was established. Then enriched for the function of related genes. The infiltration of tumors into immune cells was also analyzed, and finally, the differential expression of the four genes in different cancers was analyzed. It helps physicians in the prognosis of early LUAD patients. Furthermore, it provides some theoretical basis for personalized treatment.

## 2. Materials and Methods

**Figure 1:**
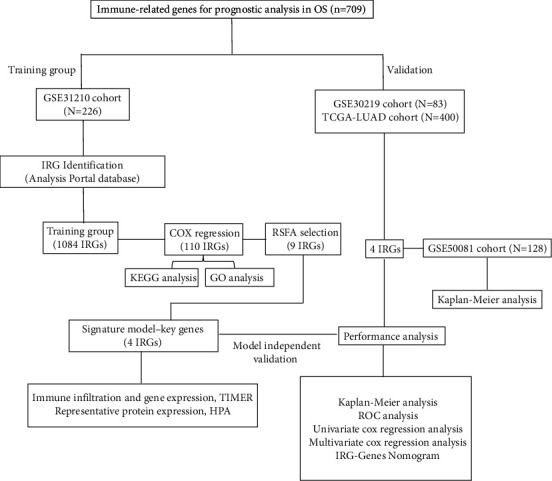
Flow chart of this study.

Figure 1 shows the flow chart of this work.

### 2.1. Expression Data

The Gene Expression Omnibus (GEO, GSE31210, GSE30219) and The Cancer Genome Atlas (TCGA) databases, which were widely used and generally recognized, were selected as the data sources for the study, from which clinical information and gene expression profiles of lung adenocarcinoma patients. GSE31210 were selected as training and GSE30219 and TCGA were selected as external validation sets, respectively. The total number of patients of the three cohorts was 709. The distribution of cases in the three data was training set GSE31210 (*N* = 226) and the validation sets are GSE30219 (*N* = 83) and TCGA (*N* = 400), respectively. Clinical information and gene expression profiles related to the dataset were collected according to the following methods. In addition, the GSE50081 (*N* = 128) dataset was also collected for the validation of the Kaplan–Meier analysis. In addition, the GPL570 microarray platform was annotated by probes to obtain the final expression profiles of the GEO data [13].

### 2.2. Selection of IRGs set

Immunology Database and Analysis Portal (ImmPort) is an open repository of discipline-level human immunology databases for translation and clinical research. The IRGs set was obtained from ImmPort, and the related *R* package “clusterProfiler” was used to match the corresponding ensemble and other related information. Finally, 1455 IRGs were obtained.

### 2.3. Development of the Prognostic Gene Signature

Prognostic immunity-related genes were obtained by univariate Cox regression screening on survival status for each gene in the training set. Genes that showed significant differences (*P* < 0.01) in the Cox regression were subsequently analyzed using the stochastic survival forest algorithm (RSFA) for dimensionality reduction [14]. In addition, the equation is shown below:

(1) $$\begin{array}{c} \mathrm{Risk}\;\mathrm{Score}=\sum_{i=1}^{N}\left(\mathrm{Exp}r_{i}+\mathrm{coef}f_{i}\right), \end{array}$$

The meaning of each parameter is as follows: in which N is the amount of IRGs, Expr is the IRGs expression value, and coeff is the coefficient value of IRGs. In the following section, patients are classified into low and high-score-risk groups according to the median value.

We combined the genes generated in RSFA with sufficient scale to prevent overfitting during modeling. The obtained combinations of IRGs were tested for their performance using time-correlated receiver operating characteristic (time-ROC) analysis. The gene combinations with the largest area under the curve (AUC) were used for subsequent predictions, and the model is statistically significant. The accuracy of the model for prognosis was subsequently validated in internal and external validation.

Clinical information about cancer patients in the dataset may also be an influential factor in prognosis. So we analyzed the relationship between the two in the following way. Evaluation of patient prognostic outcomes and their relationship to clinical characteristics Chi-square analysis was used to determine the association between the IRGs prediction model and clinical information. KM survival curves and log-rank test were used to characterize the interaction between the model and survival time. In addition, the clinical information in the training and validation sets was related to OS using multivariate Cox regression analysis. The final univariate Cox analysis was utilized to determine whether clinical features could assist immune-related gene models used together for prognosis.

### 2.4. Construction and Evaluation of an IRG Nomogram

A predictive nomogram for gene expression correlation was developed. Subsequently, we used the calibration curve to detect its accuracy in the GEO and TCGA cohorts. In addition, the prediction bias of the nomogram was assessed for evaluation.

### 2.5. Detection of the Infiltration of Immune Cells with Prognosis-Related Irgs in Tumors

Tumor Immune Estimation Resource (TIMER) is a website that uses gene expression profiling data to detect the infiltration of immune cells in tumor tissue. We applied it to explore the infiltration of immune cells with the four genes (HMOX1, ARRB1, ADM, and PDIA3) in LUAD and six infiltrating immune cells (B cells, CD4+ T cells, CD8+ T cells, neutrophils, macrophages, and dendritic cells) were available for analysis [9, 15].

### 2.6. External Validation of the IRGs Signature

Furthermore, TIMER database was utilized to validate the differential expression of the four prognostic IRGs in different cancers. The Human Protein Atlas (HPA) is based on proteomic, transcriptomic, and systems biology data and covers protein expression not only in tumors but also in normal tissues. It was used to verify the protein levels in the model.

### 2.7. Functional Enrichment Analysis

Gene Ontology (GO) enrichment and Kyoto Encyclopedia of Genes and Genomes (KEGG) pathway analysis were applied using the cluster-Profiler package to analyze the underlying biological processes of the prognostic IRGs [16].

### 2.8. Statistical Analysis

Data analysis was performed by *R* (version 4.1.2). KM and cox analyses were applied in three separate datasets using the *R* package “survival.” Cox analysis was utilized to identify prognostic IRGs and detect the models. Chi-square or rank-sum tests were applied to stratified variables. “ROC” and “TimeROC” can be used to validate the model's viability. Functional annotation is performed using the “Clusterprofiler” package. The *P*-values involved in the analysis were  < 0.05, which was a significant statistical value.

## 3. Results

### 3.1. Patient Population Information

The number of LUAD patients was obtained from GSE31210 and GSE30219, which were 226 and 83, respectively, and 400 LUAD patients were gathered from the TCGA database. A sum of 1084 IRGs was identified for expression in the GSE31210 dataset. In Table 1, we can easily obtain the median age of the patients as 61 years. The number of male and female cases in the training set was 105 and 121, respectively, with 35 deaths and 191 living cases, and the median OS was 5.33 years. Data per sample from GSE31210 and TCGA data were distributed in pathological stages I-II of LUAD, and data per sample from GSE30219 data were distributed in *T* stages T1-T2 of LUAD.

### 3.2. Identifying four Prognostic IRGs in the Training Set

**Figure 2:**
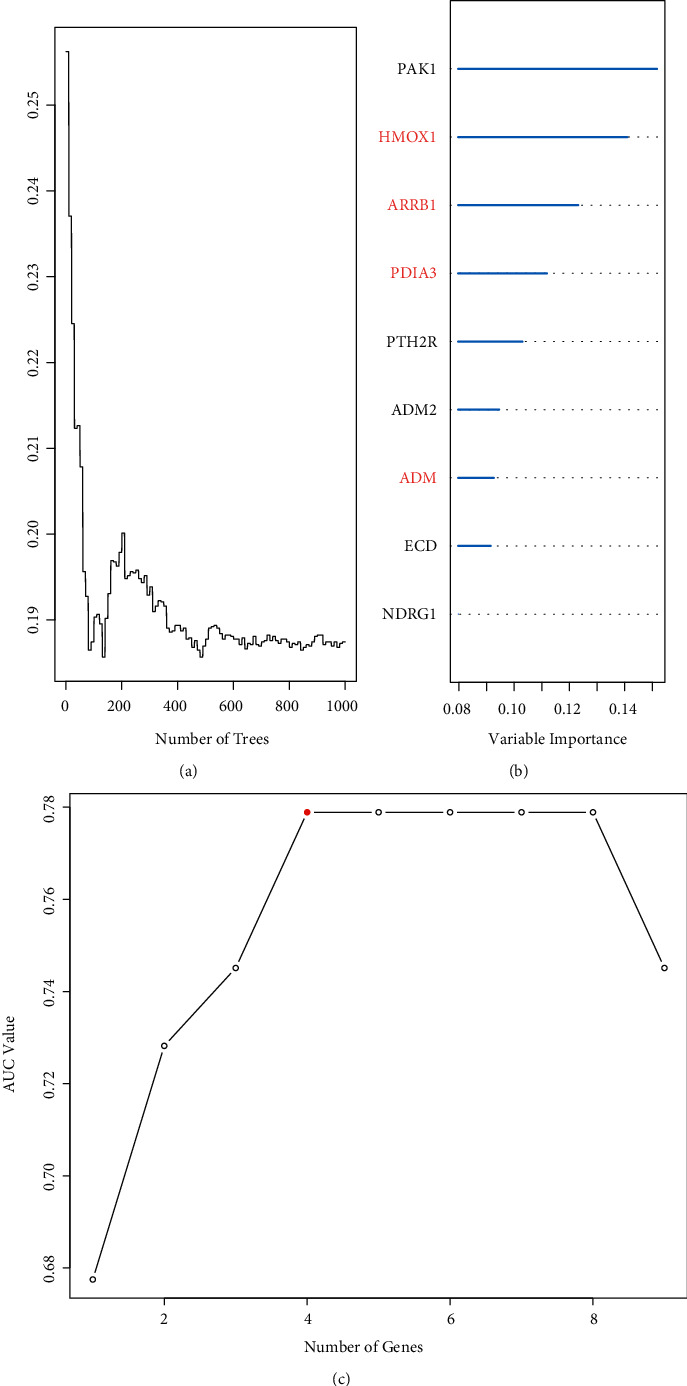
Prognosis prediction. (a, b) Selection of prognosis-related IRGs using RSFA. (c) AUC values of different IRGs prognostic models.

After univariate cox analysis and ROC curve analysis, 110 prognosis-related genes were obtained by screening based on *P* < 0.01 and AUC > 0.6, and the results are shown in Supplementary Table S1. Nine IRGs were next obtained by importance scoring in a randomized survival forest, and subsequent permutations of these nine genes yielded 2^9^–1 = 511 prediction models (Figure 2(a) and 2)(b). Then, these models were evaluated by AUC, and the combination of four genes, HMOX1, ARRB1, ADM, and PDIA3, was found to have the most considerable AUC value of 0.779 (Figure 2)(c), which had the optimal predictive power. The risk score was shown in the following formula:

(2) $$\begin{array}{c} \mathrm{Risk}\;\mathrm{Score}=\left(1.32004\times \mathrm{HMOX}1\right)+\left(-1.36259\times \mathrm{ARRB}1\right)+\left(1.17434\times \mathrm{ADM}\right)+\left(1.44023\times \mathrm{PDIA3}\right) \end{array}$$

where the gene name represents the expression level of this gene in a particular sample. From the equation, we can get the coefficients of 1.32004, −1.36259, 1.17434, and 1.44023 for HMOX1, ARRB1, ADM, and PDIA3, respectively.

**Figure 3:**
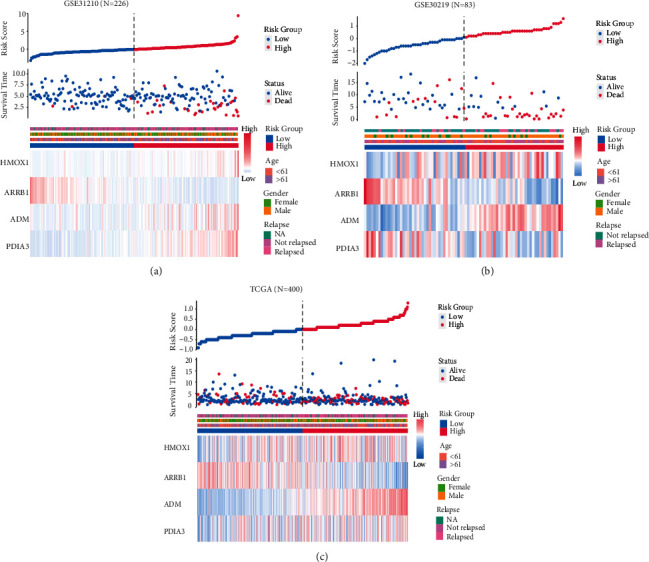
Evaluation of the risk predictive model. (a–c) Clustering Heatmap, the scatter plot of survival time, and the risk score curve of GSE31210, GSE30219, and TCGA sets. Gene expression levels, survival information, and clinical information are given.

We computed the risk scores for each patient using the RSF formula and plotted heat maps for the four genes (Figures 3(a)–3)(c). The number of deaths increased with the increase of risk score in the three datasets. In the high-risk-score group, two genes, HMOX1, ADM, were highly expressed and ARRB1 was lowly expressed, with the same pattern in all three data sets. However, there was no significant pattern in the expression of the PDIA3 gene. Among the relevant clinical features, relapses become more frequent as risk scores increase, but no significant trends were found for age and gender following the score during the three cohort. By cox analysis, ARRB1 was highly expressed in the low-risk-score group, which was a protective factor (Table 2).

### 3.3. Evaluation of a Prognostic IRGs Signature

**Figure 4:**
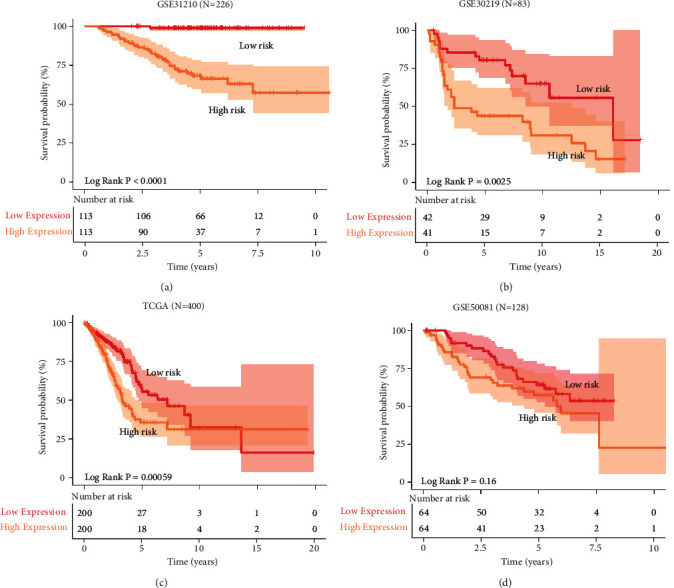
Immune-related gene modeling predicts overall survival in patients with LUAD. (a–d) Kaplan–Meier survival curves in the GSE31210, GSE30219, GSE50081, and TCGA. *P* values were calculated by log-rank test.

A risk score was computed for each individual with the prognostic models of immune-related genes. In the training cohort, the KM analysis was used to confirm the difference in survival between the high-risk-score (*N* = 113) and low-risk-score (*N* = 113) populations classified by a median score [17, 18]. In Figure 4(a), the 5-year survival incidence was 58.07% (*N* = 66) in the low -risk-score group vs. 32.74% (*N* = 37) in the high-risk-score group. In the training cohort, significant OS was observed in the low-risk-score group. To explore this in the validation set, the same methodology was then adopted for the GSE30219 (Figure 4)(b), and the model showed significant differentiation capability (the 5-year survival rate was 69.05% in the low-risk-score group (*N* = 42) vs. 36.59% in the high-score-risk group (*N* = 41), log rank *P*=0.0025). In Figure 4(c), TCGA data (*N* = 400) with a large sample size was used for survival prediction. LUAD in this cohort was also classified into high- or low-risk-score groups (the 5-year survival incidence was 13.50% in the low-risk-score group (*N* = 200) vs. 9.00% in the high-risk-score group (*N* = 200), log rank *P*=0.0025). In Figure 4(d), additional selected GSE50081 data were used for the validation of the survival model (the 5-year survival incidence was 50.00% in the low-score-risk group (*N* = 64) vs. 35.94% in the high-score-risk group (*N* = 64), log rank *P*=0.16). Although not significant, it still has some predictive power. From the results of the four cohorts, it can be obtained that the four prognostic IRGs model had excellent prognostic ability.

### 3.4. Correlation Analysis Between Signature and Clinical Features

In Table 3, the association between the IRGs signature and clinical features in the three datasets was validated by the chi-square test. There was a significant correlation between the pathological stage and the prognostic IRGs signature in the GSE31210 and TCGA cohort (*P* < 0.05). In the GSE31210 cohort, gender and age both had no obvious correlation with the prognostic IRGs signature. In the TCGA dataset, gender and the prognostic IRGs signature had a significant relationship (*P* < 0.05), but the age had not with this model. The GSE30219 cohort has a different clinical characteristic T-stage than the GSE31210 and TCGA cohort, and no statistically significant between the three clinical features and the prognostic IRGs signature in the GSE30219.

In Table 4, the IRGs signature was shown to be statistically significant by multivariate COX regression, affirming it as an independent adverse predictor. In all three data sets, signatures proved to be powerful predictors of clinical outcomes in LUAD patients (high- vs. low-risk, GSE31210, HR = 32.11, 95% CI 4.32–238.91, *P* < 0.001, *n* = 226; GSE30219, HR = 2.49, 95% CI 1.29–4.78, *P* < 0.001, *n* = 83; TCGA, HR = 1.73, 95% CI 1.18–2.54, *P* < 0.001, *n* = 400). Univariate Cox also suggests that prognostic IRGs signature is a risk factor. Interestingly, the *P*-values for the pathological stage in GSE31210 and TCGA were less than 0.5. Unfortunately, this clinical feature was not available in GSE30219, so this information could not be further judged by the three datasets. Gender and age cannot be considered as risk factor for prognosis in the three cohorts.

### 3.5. Exploring the Functions of the IRG Panel

**Figure 5:**
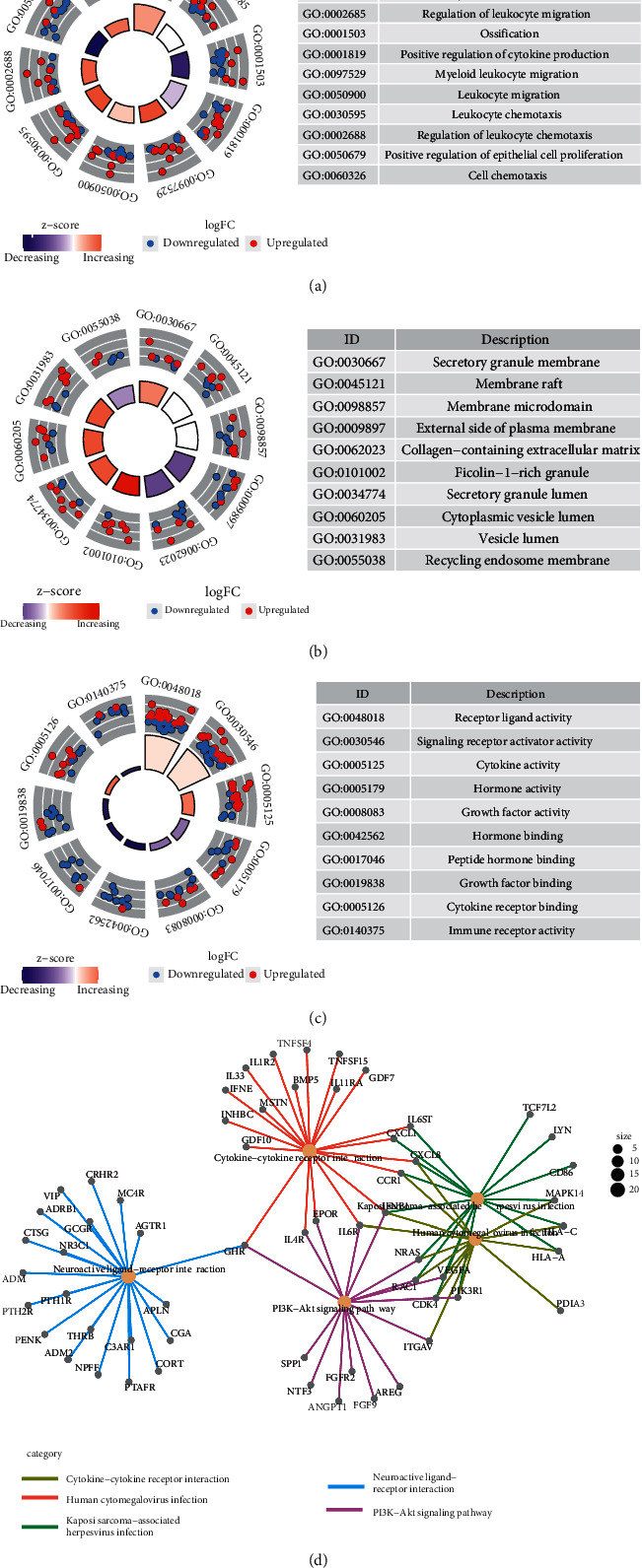
GO and KEGG analysis for the prognosis-related IRGs. (a–c) Function enrichment of biological process (BP), cellular component (CC), and molecular functions (MFs). (d) KEGG-Gene-Concept Network.

To begin with, we obtained 110 IRGs by survival analysis and AUC analysis. Then we analyzed the pathways and functions of IRGs using GO and KEGG. Top 10 functional annotations of biological processes (BPs), cellular components (CCs), and molecular functions (MFs) were selected, among which the main outcomes of BPs were regulation of chemotaxis, leukocyte migration, and cytokine production. The preliminary results of CCs were linked to the membrane. MFs enrichment analysis can be obtained concerning molecular activity and molecular binding (Figures 5(a)–5(c).

In Figure 5(d), the KEGG-Gene-Concept Network provides a clear view of the distribution of IRGs in different pathways. Most genes are associated with neuroactive ligand-receptor interactions in the KEGG-Gene-Concept Network.

### 3.6. IRGs Survival Prediction Nomogram

**Figure 6:**
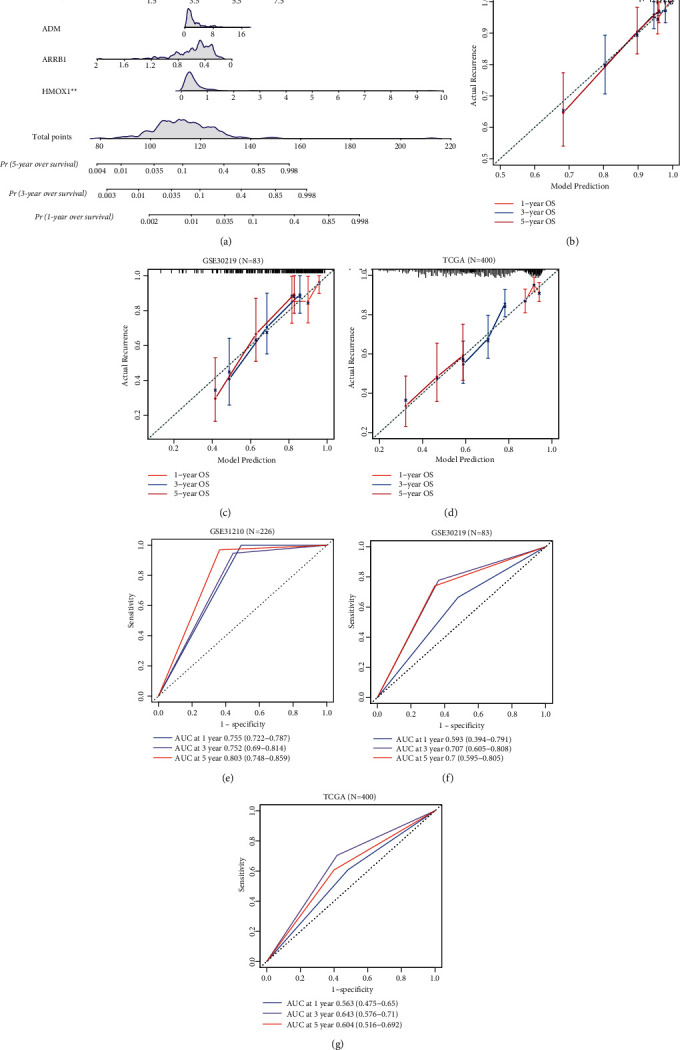
The nomogram including IRGs factor. (a) Nomogram for survival time. (b–d) Calibration curves of GSE31210, GSE30219, and TCGA. (E-G) Time-ROC curves of GSE31210, GSE30219, and TCGA.

In Figure 6(a), to enable comprehensive prediction, predictive models for the four genes were translated into a nomogram to provide a visual projection of OS at 1, 3, and 5 years. For instance, different scores were obtained from the expression of the four genes, and the total scores were summed to calculate the survival rates at different years. As can be seen from the nomogram, the two genes HMOX1 and PDIA3 in the prognostic IRGs model require focused attention in the model for prediction.

To evaluate how well this nomogram simulates the real situation, calibration curves using 1000 bootstrap tests were plotted. As shown in Figure 6(b), the actual case and the predicted situation show a good agreement in the training set. Furthermore, the calibration curve still showed a good deal in the validation set (Figures 6(c) and 6)(d). These results indicate that our nomogram is a good predictor of reality, and both independent validation databases show that the nomogram has excellent utility.

In the Figure 6(e), the time-AUC values in the GSE31210 at years of 1,3 and 5 were 0.755 (95% CI: 0.722–0.787), 0.752 (95% CI: 0.690–0.814), and 0.803 (95% CI: 0.748–0.859), respectively. All three AUC values of GSE31210 were greater than 0.7, which indicates a high degree of confidence in its prediction. Other cohorts (GSE30219 and TCGA) were used to demonstrate the accuracy of OS prediction.: The time-AUCs of GSE30219 at years of 1,3 and 5 were 0.593 (95% CI: 0.394–0.791), 0.707 (95% CI: 0.605–0.808), and 0.700 (95% CI: 0.595–0.805) (Figure 6)(f). The AUCs of TCGA set at years of 1,3 and 5 were 0.563 (95% CI: 0.475–0.650), 0.643 (95% CI: 0.576–0.710), and 0.604 (95% CI: 0.516–0.692) (Figure 6)(g). The results from these two datasets show that our prognostic IRGs model has excellent prognostic accuracy.

### 3.7. Association of Infiltration of Immune Cells with Prognosis-Related IRGs in Tumors

**Figure 7:**
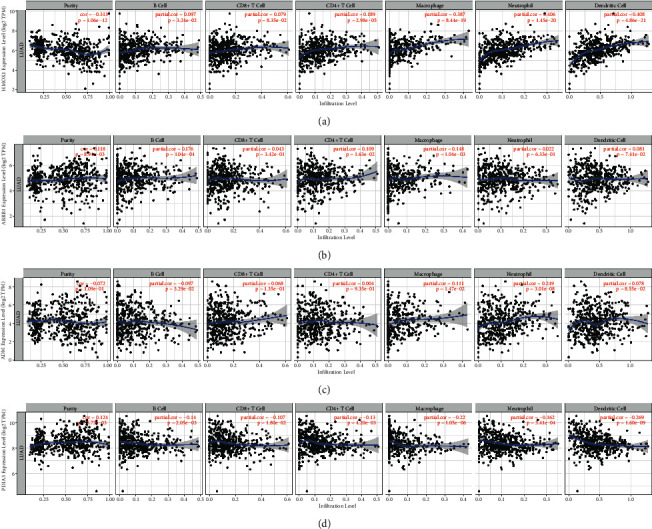
Association of infiltration of immune cells with prognosis-related IRGs in LUAD. (a) HMOX1. (b) ARRB1. (c) ADM. (d) PDIA3. *P* < 0.05 is significant. Each point means a case in the GSE31210 dataset.

The tumor microenvironment consists of stromal cells, tumor cells, and infiltrating immune cells. We utilized TIMER to explore the potential association of infiltration of immune cells with prognosis-related IRGs in tumors. Interestingly, HMOX1, ARRB1, ADM, and PDIA3 were positively associated with B cells and macrophages (*P* < 0.05). These results validate that the four IRGs are closely associated with the level of B cells and macrophages infiltration. Conversely, there is no significant association between these four genes and infiltration of purity, CD8+ T cells, CD4+ T cells, neutrophils, and dendritic cells (Figure 7(a)–7(d)).

### 3.8. IRGs Expression and Immunohistochemistry in Online Databases

**Figure 8:**
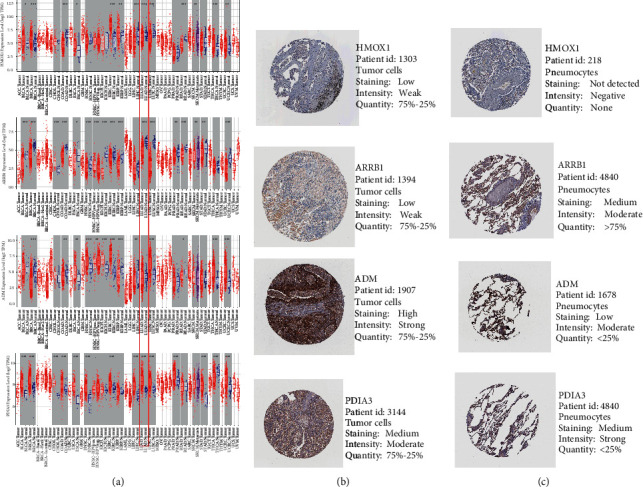
Expression of the four predictive genes. (a) HMOX1, ARRB1, ADM, and PDIA3 expression level in LUAD-tumor vs. LUAD-normal in TIMER (https://cistrome.shinyapps.io/timer/). (b) Expression of four IRGs-encoded proteins in tumor cells and normal pneumocytes in the HPA database.

In Figure 8(a), PDIA3 was highly expressed, and the results were consistent with the training set and TCGA set. ARRB1 was lowly expressed, and the results were consistent with the three independent data sets, interestingly, in LUAD, statistically significant differences in the expression of HMOX1, ARRB1, and PDIA3. However, ADM were not differentially expressed between LUAD-normal and LUAD-cancer. The differential expression levels of HMOX1, ARRB1, and PDIA3 in the prognostic IRGs model in LUAD also provide positive evidence that the model and LUAD have a strong correlation and can be used effectively for prognosis.

According to the immunohistochemical analyses in the HPA database, representative protein expressions of HMOX1, ARRB1, ADM, and PDIA3 in normal and cancerous tissues were compared (Figures 8(b) and 8)(c). The staining intensity of these two genes (HMOX1, ADM) in tumor cells is higher than in normal pneumocytes. The staining intensity of ARRB1 in tumor cells is lower than in normal pneumocytes, while PDIA3 did not show a striking difference.

## 4. Discussion

When immunotherapy was not well established, early treatment of LUAD was importantly based on surgical lobectomy, with suboptimal outcomes because of the malignant nature of LUAD and the limited results of surgery. However, with the increasing maturity and clinical use of immune checkpoint inhibitors and targeted therapies, the prognosis of patients has improved significantly [19, 20]. In this study, we choose three separate datasets, GSE31210, GSE30219, and TCGA, for the establishment and validation of an IRGs prognostic model. First, we carried out a dimensionality reduction on the obtained IRGs and subsequently avoided overfitting the model. Finally, we obtained 511 combinations by recombination and performed screening to obtain four gene prediction signatures.

Several recent studies have used IRG characteristics to predict the prognosis of LUAD patients. However, not all models showed an excellent prognosis. Wu et al. developed a 21-IRGs prognostic model. In this study, multivariate regression was used to screen for genes of relevance, but there is no practical method to prevent overfitting. Unfortunately, the AUC was only 0.61, 0.65, and 0.62 for 1, 3, and 5-year survival in the training set [21]. While the present study avoided overfitting and predicted 1, 3, and 5-year survival by four immune-related genes models, the AUC obtained was 0.755, 0.752, and 0.803 in the training set.

The included IRGs for signature were HMOX1, ARRB1, ADM, and PDIA3. HMOX1 (Heme Oxygenase 1) is a protein-coding gene whose related functions include protein homodimerization activity and oxidoreductase activity [22]. Several published pieces of evidence support the overexpression of HMOX-1 in several human malignant tumors, including kidney, gastrointestinal, lung, and breast cancers [23]. Tsai et al. research discovered that HMOX1 is a negative prognostic NSCLC gene. Its high expression may increase the metastasis ability of cancer cells of NSCLC patients, and HMOX1 has the potential as a therapeutic target for NSCLC in the future [24].

ARRB1 (Arrestin Beta 1) plays an important role in transcription factor binding and ubiquitin protein ligase binding functions, and it is also a protein-coding gene [25]. Li et al. suggest that ARRB1 may be associated with the prognosis of LUAD, and it is considered as a new molecular biomarker for the diagnosis and prognosis of LUAD [26].

ADM (Adrenomedullin) gene encodes a protein that is a prohormone with several functions, including vasodilation, hormone secretion regulation, angiogenesis promotion, and antimicrobial activity [27]. Some studies suggest that NSCLC cells are essential targets for ADM, and it may regulate the activity of these malignant lung cells through differential induction of different early response genes [28].

PDIA3 (Protein disulfide isomerase A3) gene encodes a protein that is a member of the PDI family. PDIA3 has high expression levels in response to cellular stress and blocks apoptotic cell death associated with endoplasmic reticulum (ER) stress and protein misfolding. PDIA3 is highly expressed in most cancers, and its expression is associated with overall low cell survival, metastasis, and invasiveness [29]. Wang et al. demonstrated the potential prognostic value of PDIA3 through a proteomic biomarker discovery approach [30].

Using nomogram to show the prediction of survival by IRGs signatures, it visually exhibits excellent prognostic capability. The calibration curves also show good agreement between the predictions value and the actual situation in training. We also present the cellular functions and pathway enrichment of the selected IRGs based on GO analysis and KEGG analysis. According to GO analysis, regulation of chemotaxis, leukocyte migration, and cytokine production were significantly enriched functions and most genes associated with neuroactive ligand-receptor interactions based on KEGG-Gene-Concept Network.

By exploring the potential association of infiltration of immune cells with prognosis-related IRGs in tumors, we found that the four IRGs were closely associated with the level of B-cell and macrophage infiltration. HMOX1, ARRB1, and PDIA3 were significantly differentially expressed in LUAD-normal and LUAD-cancer. The proteins encoded by the three genes HMOX1, ADM, and ARRB1 also differ significantly in tumor cells and normal pneumocytes. These results suggest that the four IRGs have prognostic potential.

This study also has some restrictions. Firstly, the cohorts of GEO and TCGA databases we used for the construction of immune-related gene prognostic models are retrospective experiments and lack large clinical samples to prospectively validate the prognostic value of LUAD patients. Additionally, due to the lack of experimental exploration in these immune genes' potential functions and mechanisms, the potential functions and mechanisms of these immune genes need to be further validated in clinical trials.

## 5. Conclusion

In summary, our study systematically analyzed the expression, prognostic value, and potential functions of IRGs. Ultimately, a four-immune-related genes signature was constructed that can be independently used as a biomarker for OS prediction in LUAD. Eventually, our study provides an essential theoretical basis for further research on the role of IRGs in LUAD.

## Figures and Tables

**Table 1:** Clinical information of the Gene Expression Omnibus (GEO) and The Cancer Genome Atlas (TCGA) datasets.

Characteristic	GSE31210	GSE30219	TCGA
Age (years)			
>61	122	37	251
≤61	104	46	149
Sex			
Female	121	65	217
Male	105	18	183
Vital status			
Alive	191	39	278
Dead	35	42	122
Pathological stage			
Stage I	168	—	280
Stage II	58	—	120
T stage			
T1	—	69	—
T2	—	12	—

**Table 2:** Prognosis of the four genes in the signature.

ENSEMBL ID	Symbol ID	Gene name	Coef	*P*-value	Prognostic indicator
ENSG00000100292	HMOX1	Heme Oxygenase 1	1.32	<0.01	high
ENSG00000137486	ARRB1	Arrestin Beta 1	−1.36	<0.01	low
ENSG00000148926	ADM	Adrenomedullin	1.17	<0.01	high
ENSG00000167004	PDIA3	Protein Disulfide Isomerase Family A Member 3	1.44	<0.01	high

**Table 3:** The IRG signature and clinical characteristics Chi-square table in LUAD patients.

Variables	Status	low	high	*P*
*GSE31210 dataset (N* *=* *226)*				
Age				0.69
	≤61	63	59	
	>61	50	54	
Gender				0.11
	Female	67	54	
	Male	46	59	
Pathological stage				<0.01
	I	103	65	
	II	10	48	

*GSE30219 dataset (N* *=* *83)*				
Age				1.00
	≤61	23	22	
	>61	18	18	
Gender				0.30
	Female	11	6	
	Male	30	34	
T stage				0.32
	T1	37	32	
	T2	4	8	

*TCGA dataset (N* *=* *400)*				
Age				0.62
	≤61	67	73	
	>61	128	123	
Gender				0.04
	Female	119	98	
	Male	81	102	
Pathological stage				0.02
	I	151	129	
	II	49	71	

**Table 4:** Cox regression analysis of the IRG signature and clinical information with lung adenocarcinoma (LUAD) survival.

Variables		Univariable cox				Multivariable cox			
		HR	95% CI of HR		*P*	HR	95% CI of HR		*P*
			right	left			right	left	
*GSE31210 (N* *=* *226)*									
Age	>61 vs. ≤61	1.4	0.73	2.78	0.29	1.38	0.7	2.71	0.35
Sex	Male vs. Female	1.52	0.78	2.96	0.22	1.22	0.63	2.39	0.56
Pathological stage	II vs. I	4.23	2.17	8.23	<0.01	2.07	1.04	4.1	0.03
Signature	High risk vs. low risk	42.31	5.79	309.3	<0.01	32.11	4.32	238.91	<0.01

*GSE30219 (N* *=* *83)*									
Age	>61 vs. ≤61	1.63	0.88	3.01	0.12	1.55	0.83	2.88	0.17
Sex	Male vs. Female	1.37	0.61	3.09	0.44	1.11	0.49	2.56	0.8
T stage	T2 vs. T1	2.14	1.06	4.32	0.03	1.68	0.82	3.42	0.15
Signature	High risk vs. Low risk	2.65	1.39	5.06	<0.01	2.49	1.29	4.78	<0.01

*TCGA (N* *=* *400)*									
Age	>61 vs. ≤61	1.07	0.73	1.56	0.73	1.18	0.8	1.73	0.4
Sex	Male vs. Female	1.03	0.72	1.47	0.87	0.92	0.63	1.33	0.64
Pathological stage	II vs. I	2.48	1.73	3.57	<0.01	2.2	1.52	3.18	<0.01
Signature	High risk vs. Low risk	1.88	1.3	2.7	<0.01	1.73	1.18	2.54	<0.01

## Data Availability

https://www.ncbi.nlm.nih.gov/geo/, https://portal.gdc.cancer.gov/

## Supplementary Materials

Supplementary Materials After univariate cox analysis and ROC curve analysis, 110 prognosis-related genes were obtained by screening based on *P* < 0.01 and AUC > 0.6, and the results are shown in Supplementary Table S1. Supplementary Table S1 |m6A gene sets of Univariate Cox regression analysis in the GSE31210 set (*P* < 0.01, AUC > 0.6, *n* = 226).
