# Supplementary material for: Assessing the Prognostic Capability of Immune-Related Gene Scoring Systems in Lung Adenocarcinoma
Source: J Oncol. 2022 Jul 31;2022:2151396. doi: 10.1155/2022/2151396 (PMC9357717; doi:10.1155/2022/2151396)
Supplement: Supplementary Materials — After univariate cox analysis and ROC curve analysis, 110 prognosis-related genes were obtained by screening based on P < 0.01 and AUC > 0.6, and the results are shown in Supplementary Table S1. Supplementary Table S1 |m6A gene sets of Univariate Cox regression analysis in the GSE31210 set (P < 0.01, AUC > 0.6, n = 226). [file 2151396.f1.docx]

| Supplementary Table S1 \| m6A gene sets of Univariate Cox regression analysis in the GSE31210 set (P<0.01,AUC>0.6, n = 226) | | | | | | | |
| --- | --- | --- | --- | --- | --- | --- | --- |
| Genes | coefficient | cox_Pvalue | HR | right | left | KM_Pvalue | AUC |
| ENSG00000011422 | 1.53181 | 0.000293 | 4.626543 | 2.019376 | 10.59976 | 6.79E-05 | 0.677487 |
| ENSG00000043591 | -1.35096 | 0.000799 | 0.258992 | 0.117584 | 0.570461 | 0.000303 | 0.660583 |
| ENSG00000066468 | -1.05565 | 0.004871 | 0.347966 | 0.166869 | 0.725599 | 0.003217 | 0.626776 |
| ENSG00000077238 | 0.97191 | 0.009413 | 2.642988 | 1.269113 | 5.504149 | 0.006936 | 0.626776 |
| ENSG00000082175 | -0.96947 | 0.00958 | 0.379283 | 0.182147 | 0.789776 | 0.007078 | 0.626776 |
| ENSG00000089127 | 1.342543 | 0.000862 | 3.828767 | 1.738198 | 8.433706 | 0.000335 | 0.660583 |
| ENSG00000089250 | -0.98316 | 0.008724 | 0.374127 | 0.179446 | 0.780019 | 0.006363 | 0.626776 |
| ENSG00000089685 | 1.00077 | 0.007527 | 2.720376 | 1.305834 | 5.667215 | 0.005354 | 0.626776 |
| ENSG00000090006 | -1.16777 | 0.002576 | 0.311059 | 0.145569 | 0.664686 | 0.001441 | 0.64368 |
| ENSG00000100292 | 1.320041 | 0.00105 | 3.743574 | 1.699729 | 8.245046 | 0.000434 | 0.660583 |
| ENSG00000100448 | -1.08744 | 0.004972 | 0.337078 | 0.157831 | 0.719897 | 0.003195 | 0.64368 |
| ENSG00000102678 | -0.97992 | 0.008876 | 0.375341 | 0.180167 | 0.781948 | 0.006485 | 0.626776 |
| ENSG00000102908 | -1.09186 | 0.004779 | 0.335591 | 0.157187 | 0.71648 | 0.003044 | 0.64368 |
| ENSG00000104419 | 1.172899 | 0.002436 | 3.231346 | 1.513638 | 6.898347 | 0.001339 | 0.64368 |
| ENSG00000105220 | 1.355608 | 0.000763 | 3.87912 | 1.761583 | 8.542073 | 0.000285 | 0.660583 |
| ENSG00000106113 | 0.954283 | 0.009008 | 2.596809 | 1.268902 | 5.31437 | 0.006731 | 0.609873 |
| ENSG00000109321 | -1.00374 | 0.007387 | 0.366505 | 0.17585 | 0.763865 | 0.005242 | 0.626776 |
| ENSG00000110492 | 1.1124 | 0.004053 | 3.041649 | 1.424447 | 6.49489 | 0.002497 | 0.64368 |
| ENSG00000112062 | 0.991992 | 0.008056 | 2.696602 | 1.294626 | 5.616805 | 0.005793 | 0.626776 |
| ENSG00000112175 | -1.15568 | 0.002814 | 0.314843 | 0.147502 | 0.672034 | 0.001599 | 0.64368 |
| ENSG00000112715 | 1.181168 | 0.002293 | 3.258176 | 1.524982 | 6.961202 | 0.001247 | 0.64368 |
| ENSG00000112964 | -0.9991 | 0.007636 | 0.36821 | 0.17673 | 0.767151 | 0.005446 | 0.626776 |
| ENSG00000113580 | -1.19666 | 0.002007 | 0.302201 | 0.141437 | 0.645695 | 0.001058 | 0.64368 |
| ENSG00000114013 | 1.14942 | 0.002968 | 3.15636 | 1.478684 | 6.737483 | 0.001707 | 0.64368 |
| ENSG00000115590 | 1.140949 | 0.0032 | 3.129737 | 1.465741 | 6.682801 | 0.001874 | 0.64368 |
| ENSG00000117586 | 1.056914 | 0.004788 | 2.877476 | 1.380681 | 5.996944 | 0.003148 | 0.626776 |
| ENSG00000118785 | 1.568594 | 0.00021 | 4.799894 | 2.094365 | 11.00046 | 4.22E-05 | 0.677487 |
| ENSG00000120210 | 1.095245 | 0.004629 | 2.989916 | 1.400996 | 6.380889 | 0.002927 | 0.64368 |
| ENSG00000120937 | 1.13607 | 0.003322 | 3.114506 | 1.458995 | 6.648513 | 0.001959 | 0.64368 |
| ENSG00000122861 | 1.023982 | 0.006267 | 2.784259 | 1.336106 | 5.802008 | 0.004324 | 0.626776 |
| ENSG00000122882 | 1.049804 | 0.005076 | 2.857092 | 1.370966 | 5.954174 | 0.003373 | 0.626776 |
| ENSG00000128165 | 0.99407 | 0.007908 | 2.702209 | 1.297573 | 5.627377 | 0.005667 | 0.626776 |
| ENSG00000131910 | -1.06801 | 0.004342 | 0.343693 | 0.164981 | 0.715994 | 0.0028 | 0.626776 |
| ENSG00000133112 | 1.159267 | 0.002743 | 3.187596 | 1.492818 | 6.806433 | 0.001552 | 0.64368 |
| ENSG00000133116 | -1.16708 | 0.002577 | 0.311274 | 0.145733 | 0.664857 | 0.001439 | 0.64368 |
| ENSG00000134352 | -1.3579 | 0.000756 | 0.257199 | 0.116712 | 0.56679 | 0.000283 | 0.660583 |
| ENSG00000135346 | -1.10589 | 0.004258 | 0.330915 | 0.155022 | 0.706383 | 0.002648 | 0.64368 |
| ENSG00000135413 | 0.992678 | 0.008087 | 2.698453 | 1.294391 | 5.625536 | 0.005828 | 0.626776 |
| ENSG00000135446 | 1.466252 | 0.000523 | 4.332967 | 1.892122 | 9.922509 | 0.000153 | 0.677487 |
| ENSG00000136238 | 1.310366 | 0.001146 | 3.707529 | 1.6831 | 8.166936 | 0.000486 | 0.660583 |
| ENSG00000136859 | 1.043825 | 0.005345 | 2.84006 | 1.362545 | 5.919763 | 0.003587 | 0.626776 |
| ENSG00000137033 | -1.11005 | 0.004125 | 0.329542 | 0.154351 | 0.70358 | 0.00255 | 0.64368 |
| ENSG00000137070 | -1.10674 | 0.004274 | 0.330635 | 0.154753 | 0.706414 | 0.002666 | 0.64368 |
| ENSG00000137486 | -1.36258 | 0.00072 | 0.255998 | 0.11622 | 0.563887 | 0.000264 | 0.660583 |
| ENSG00000137872 | -1.13948 | 0.003224 | 0.319985 | 0.149915 | 0.682991 | 0.001889 | 0.64368 |
| ENSG00000138379 | -1.16546 | 0.002599 | 0.31178 | 0.146031 | 0.66566 | 0.001451 | 0.64368 |
| ENSG00000138448 | 1.336386 | 0.000917 | 3.805266 | 1.726678 | 8.386077 | 0.000365 | 0.660583 |
| ENSG00000138623 | 1.177376 | 0.002343 | 3.245846 | 1.52047 | 6.929116 | 0.001277 | 0.64368 |
| ENSG00000139574 | -0.94762 | 0.009575 | 0.387664 | 0.189287 | 0.793948 | 0.007228 | 0.609873 |
| ENSG00000140464 | 1.35484 | 0.000776 | 3.876141 | 1.759109 | 8.540955 | 0.000293 | 0.660583 |
| ENSG00000140564 | 0.980884 | 0.008817 | 2.666812 | 1.279987 | 5.556217 | 0.006436 | 0.626776 |
| ENSG00000142273 | 1.565573 | 0.000215 | 4.785417 | 2.088197 | 10.9665 | 4.39E-05 | 0.677487 |
| ENSG00000143869 | -1.0061 | 0.007201 | 0.365641 | 0.175545 | 0.761593 | 0.005082 | 0.626776 |
| ENSG00000144407 | 1.307299 | 0.001181 | 3.696176 | 1.677474 | 8.144219 | 0.000507 | 0.660583 |
| ENSG00000144891 | -1.46673 | 0.000521 | 0.230678 | 0.100742 | 0.528206 | 0.000151 | 0.677487 |
| ENSG00000145675 | -1.10339 | 0.00436 | 0.331744 | 0.155367 | 0.708349 | 0.002727 | 0.64368 |
| ENSG00000146232 | 1.303877 | 0.00121 | 3.683549 | 1.6724 | 8.113213 | 0.000522 | 0.660583 |
| ENSG00000146469 | -1.14037 | 0.003203 | 0.3197 | 0.14977 | 0.682432 | 0.001874 | 0.64368 |
| ENSG00000148737 | -1.16981 | 0.002505 | 0.310424 | 0.145391 | 0.662786 | 0.001387 | 0.64368 |
| ENSG00000148926 | 1.174336 | 0.002408 | 3.235995 | 1.515686 | 6.908859 | 0.001321 | 0.64368 |
| ENSG00000149269 | 1.085129 | 0.005026 | 2.959821 | 1.386797 | 6.317105 | 0.003231 | 0.64368 |
| ENSG00000149923 | 1.105158 | 0.004324 | 3.019701 | 1.413497 | 6.451089 | 0.002702 | 0.64368 |
| ENSG00000150938 | -1.26543 | 0.00169 | 0.282119 | 0.128052 | 0.621551 | 0.000802 | 0.660583 |
| ENSG00000151090 | -1.26768 | 0.001661 | 0.281484 | 0.127745 | 0.620244 | 0.000786 | 0.660583 |
| ENSG00000154188 | -1.3062 | 0.00118 | 0.270846 | 0.123013 | 0.59634 | 0.000504 | 0.660583 |
| ENSG00000158869 | 1.06076 | 0.004642 | 2.888564 | 1.385893 | 6.020527 | 0.003035 | 0.626776 |
| ENSG00000159167 | 1.522366 | 0.000324 | 4.583058 | 1.99861 | 10.50951 | 7.91E-05 | 0.677487 |
| ENSG00000160712 | -1.49597 | 0.000404 | 0.224031 | 0.0978 | 0.513187 | 0.000107 | 0.677487 |
| ENSG00000160801 | -1.01751 | 0.006571 | 0.361493 | 0.17355 | 0.752965 | 0.004566 | 0.626776 |
| ENSG00000163631 | -1.1285 | 0.003551 | 0.323518 | 0.151504 | 0.690834 | 0.002128 | 0.64368 |
| ENSG00000163739 | 1.279416 | 0.001487 | 3.594538 | 1.632621 | 7.914088 | 0.000679 | 0.660583 |
| ENSG00000163823 | 1.035579 | 0.005679 | 2.816736 | 1.352165 | 5.867632 | 0.003848 | 0.626776 |
| ENSG00000164733 | 1.35166 | 0.00079 | 3.863833 | 1.754733 | 8.507963 | 0.000298 | 0.660583 |
| ENSG00000166603 | -0.99236 | 0.008036 | 0.370702 | 0.177967 | 0.772165 | 0.005776 | 0.626776 |
| ENSG00000166736 | 1.314974 | 0.001094 | 3.724655 | 1.691495 | 8.201653 | 0.000457 | 0.660583 |
| ENSG00000166923 | 1.070526 | 0.004272 | 2.916913 | 1.399643 | 6.078967 | 0.002748 | 0.626776 |
| ENSG00000167004 | 1.440234 | 0.000658 | 4.221685 | 1.843337 | 9.668671 | 0.000209 | 0.677487 |
| ENSG00000169403 | 0.97098 | 0.0095 | 2.640531 | 1.267693 | 5.500076 | 0.007013 | 0.626776 |
| ENSG00000169418 | -1.43891 | 0.000669 | 0.237186 | 0.103528 | 0.543399 | 0.000215 | 0.677487 |
| ENSG00000169429 | 0.986315 | 0.008417 | 2.681335 | 1.287414 | 5.584497 | 0.006095 | 0.626776 |
| ENSG00000170458 | 1.001951 | 0.007432 | 2.72359 | 1.307783 | 5.672151 | 0.005271 | 0.626776 |
| ENSG00000171388 | 1.024158 | 0.006231 | 2.78475 | 1.336851 | 5.800824 | 0.004291 | 0.626776 |
| ENSG00000171855 | -0.99861 | 0.007637 | 0.368392 | 0.176881 | 0.767257 | 0.005442 | 0.626776 |
| ENSG00000171860 | 1.185701 | 0.002186 | 3.272979 | 1.532935 | 6.988157 | 0.001172 | 0.64368 |
| ENSG00000175189 | -0.97436 | 0.009233 | 0.377435 | 0.181241 | 0.786012 | 0.006784 | 0.626776 |
| ENSG00000175745 | -1.11526 | 0.003964 | 0.327831 | 0.153511 | 0.700099 | 0.002432 | 0.64368 |
| ENSG00000177463 | -1.0306 | 0.00594 | 0.356794 | 0.171216 | 0.74352 | 0.00406 | 0.626776 |
| ENSG00000181195 | -1.14162 | 0.00319 | 0.319301 | 0.149507 | 0.68193 | 0.001868 | 0.64368 |
| ENSG00000181634 | -1.14176 | 0.003214 | 0.319256 | 0.149383 | 0.682302 | 0.001889 | 0.64368 |
| ENSG00000183486 | 1.148332 | 0.003003 | 3.152931 | 1.476805 | 6.731407 | 0.001733 | 0.64368 |
| ENSG00000184009 | 1.131833 | 0.003435 | 3.101336 | 1.453015 | 6.61954 | 0.002041 | 0.64368 |
| ENSG00000184995 | 0.949868 | 0.009269 | 2.585367 | 1.26411 | 5.287614 | 0.00695 | 0.609873 |
| ENSG00000185008 | -1.12964 | 0.003516 | 0.323149 | 0.151338 | 0.690014 | 0.002102 | 0.64368 |
| ENSG00000185033 | 1.500867 | 0.000387 | 4.485575 | 1.958116 | 10.27538 | 0.0001 | 0.677487 |
| ENSG00000185652 | -1.2532 | 0.001854 | 0.28559 | 0.129728 | 0.628713 | 0.0009 | 0.660583 |
| ENSG00000186868 | -1.17514 | 0.002396 | 0.308777 | 0.144607 | 0.659323 | 0.001314 | 0.64368 |
| ENSG00000187266 | -1.26598 | 0.001708 | 0.281962 | 0.127839 | 0.621896 | 0.000816 | 0.660583 |
| ENSG00000196262 | 1.751084 | 9.58E-05 | 5.760845 | 2.390003 | 13.8859 | 1.01E-05 | 0.69439 |
| ENSG00000204351 | 1.034506 | 0.00572 | 2.813717 | 1.350915 | 5.860475 | 0.003878 | 0.626776 |
| ENSG00000204525 | 1.055098 | 0.004923 | 2.872258 | 1.376709 | 5.992451 | 0.003262 | 0.626776 |
| ENSG00000205213 | -1.42097 | 0.000775 | 0.24148 | 0.105452 | 0.552981 | 0.000262 | 0.677487 |
| ENSG00000206503 | 1.034049 | 0.005893 | 2.812431 | 1.347239 | 5.871097 | 0.004037 | 0.626776 |
| ENSG00000211672 | 1.071161 | 0.004261 | 2.918765 | 1.400216 | 6.084194 | 0.002742 | 0.626776 |
| ENSG00000213281 | 1.531203 | 0.000299 | 4.623734 | 2.016405 | 10.60249 | 7.05E-05 | 0.677487 |
| ENSG00000213658 | -1.01146 | 0.006943 | 0.363688 | 0.174504 | 0.757971 | 0.004876 | 0.626776 |
| ENSG00000215644 | -0.98996 | 0.008176 | 0.37159 | 0.178421 | 0.773895 | 0.005891 | 0.626776 |
| ENSG00000240972 | 1.325303 | 0.001002 | 3.763325 | 1.708716 | 8.288456 | 0.000408 | 0.660583 |
| ENSG00000241563 | -1.032 | 0.005885 | 0.356293 | 0.170944 | 0.742611 | 0.004017 | 0.626776 |
| ENSG00000254087 | 1.13672 | 0.003319 | 3.116529 | 1.459388 | 6.65536 | 0.00196 | 0.64368 |
| ENSG00000266524 | -1.29602 | 0.001295 | 0.273619 | 0.124231 | 0.602645 | 0.00057 | 0.660583 |
